# Forensic Odontology: The New Dimension in Dental Analysis

**Published:** 2017-03

**Authors:** K. P. Divakar

**Affiliations:** Lecturer, Department of Conservative Dentistry and Endodontics, DAPMRV Dental College, CA-37, 24^th^ Main, 1 Phase J.P. Nagar, Bangalore, Karnataka, India

**Keywords:** Forensic odontology, DNA Analysis, Bite Marks

## Abstract

Forensic Odontology a branch of Forensic sciences uses the skill of the dentist in personal identification during mass calamities, sexual assault and child abuse to name a few. This branch not stranger to many has been growing tenfold in its potential and its ability to bring the forlorn to justice where a dental remains is the only available evidence. It’s role and importance in the judiciary is fast growing and hence in depth knowledge in this field seems more than justified.

## INTRODUCTION

Forensic odontology is proper handling, examination, and evaluation of dental evidence, which will be presented in the interest of justice. The evidence that may be derived from the teeth, the age (in children) and identification of the person to whom the teeth may belong. Knowledge of forensic dentist requires encompassing of number of disciplines, since the dental records obtained can identify an individual or afford the information needed by the authorities to establish identification of the case (1).

Keiser-Neilson defined forensic dentistry as “that branch of forensic dentistry that in the interest of justice deals with the proper handling and examination of dental evidence and the proper evaluation and presentation of dental findings (2)”.

This article will give you a collective review on the evolution, various methods and applications of forensic odontology, which plays a major role in keeping the dental records accurately, and providing required information, which will help the legal authorities to recognize negligence, malpractice, fraud or abuse and identification of unknown individuals.

## HISTORICAL BACKGROUND

66 AD: Well-documented evidence to the use of teeth for identification began AD with Agrippina and Lollia Pauline case. It was the first use of dental identification where there is a record.

1193: The first forensic identification in India started in were Jai Chand, a great Indian monarchy was destroyed by Muhammad’s army and Jai Chand, Raja of Kanauji was murdered and he was identified by his false teeth.

1758: Peter Halket was killed in during French and Indian wars in a battle near Fort Duquesne. Halket son identified his father’s skeleton by an artificial tooth.

1776: At the battle for Breed’s Hill in Boston, Dr. Joseph Waren was killed in the year His face was not able to identify as he suffered from a fatal head wound. A dentist, Paul Revere, identified Dr. Warren, dead body by a small denture that he had fabricated for him.

## IDENTIFICATION UNKNOWN REMAINS

Dental identification plays an important role when identification of remains of deceased person is skeletonized, decomposed, burned or dismembered and is invalid by visual or fingerprint methods.

The identification of remains by dental evidence is possible because, the hard tissues are preserved after death and can even withstand a temperature of 1600 degree C when heated without appreciable loss of microstructure, and the status of a person’s teeth change throughout the life and the combination of decay, missing, filling can be obtained from any fixed time (2).

Odontological identification is based on systemic comparison of pre and post mortem dental characteristics of individual based on dental records and the supporting radiographs (3). But this technique is complicated by the trauma to jaws and inadequate ante-mortem dental information.

According to American board of forensic odontology dental identification can be divided into four types (3):

1. Positive identification: The ante-mortem and postmortem data match to establish that it is from same individual;
2. Possible identification: The ante-mortem and postmortem data have few consistent features, but because of quality of the records it is difficulty to establish the identity;
3. Insufficient evidence: The data is not enough to from the conclusion;
4. Exclusion: The ante-mortem and postmortem data clearly inconsistent.

In many cases, dental identification, fingerprint, and DNA are most commonly used and often complement each other.

## DETERMINATION OF SPECIES

It comes into picture when the evidence found at the scene of crime is a fragment of mandible or just a single tooth, which is of few millimeters in size (5). It is possible to determine species by dental tissues because the dentinal fluids contain special information, which can be compared and analyzed using counter current electrophoresis with artificially antisera (3).

## DNA ANALYSIS IN FORENSIC DENTISTRY

The sample for DNA analysis is isolated from the biological material such as blood, semen, hair roots, tissue, teeth, bone and saliva (5).

## SPECIMEN SELECTION

In the decomposed post-mortem tissue, Although DNA undergoes progressive fragmentation through autolytic and bacterial enzymes; the sequence of information is still present in the DNA fragment (7). Therefore information is not completely lost even though the body has undergone decomposition. In the fresh cadaver, unclotted blood can be source of DNA (8).

### Storage

The specimen should be stored in cold place or frozen. Desiccation, simple air-drying can be used to store bone and bloodstains. Tissue in formalin is often used for PCR based DNA testing^9^.

### Collection

One should be very careful about contaminating the specimens so should wear gloves and use pristine instruments. The collection of fresh specimen is done by incisional biopsy (9).

### Reference sample

When there is no much information about ante-mortem of the individual, the specimens can be selected from spouse and children as reference sample for DNA testing. Forensic odontology has an important role because teeth and saliva is excellent source of DNA. Since 1992, the isolation of DNA from saliva and salivary stained material is done (9).

### Saliva

It is major source of DNA because; it contains sloughed epithelial cells from oral mucosa and inner surface of lip. The enzymes such as Streptococcus Salivarius and Streptococcus Mutans are present on teeth and in the saliva (11). In Polymerase chain reaction (PCR) technology, the Streptococcal DNA sequence provides a means with which to identify the bacterial composition from bite marks and can be matched exclusively to those from the teeth responsible (9).

### Teeth

Teeth with act as major source of DNA because its ability to withstand to undergo changes. Some authors suggest that teeth are better sources of DNA than skeleton bones. DNA is found in vascular pulp, odontoblastic process, accessory canals, and cellular cementum (14).

Dental pulp can also be used for DNA analysis and is good source for determination of blood groups. The presence of ABO blood grouping antigens in soft and hard tissues make possible to determine blood group of highly decomposed remains (17). The DNA from the teeth is not only acts for primary identification but it can also be used as reference sample to relate the other tissue fragments.

## FACIAL RECONSTRUCTION AND FACIAL SUPERIMPOSITION

The technique where face is constructed on bare bone of the skull. In most of cases the post-mortem profile does not elicit the identity of the deceased so it may be important to reconstruct the individual’s appearance during life (11). Forensic artist utilizes the ante mortem photograph of dental profile to help in facial reconstruction.

In most of cases the clues found on the crime site can be helpful to construct the face like dress size, other clothing may indicate the gender of the individual, hair may be present, skin tag to determine the color and racial species (18).

Eyeglasses, hearing aids and condition of teeth can give idea about the age of the patient. But this technique requires the availability of suitable ante-mortem photographs showing the teeth. Many authors suggest this technique as under development, unscientific and unacceptable. But it is surely another tool for identification (10).

## AGE ESTIMATION BASED ON DENTAL DATA

The need for age estimation has increased in recent years because there is increase in numbers of unidentified cadavers and human remain especially in metropolitan cities and age estimation for living individuals who do not have valid proof of date of birth with them (9).

Dental ageing technique can be broken down into two categories (10).

1. Developmental changes: Developmental changes that occur to the human dentition while the teeth are growing and emerging into the oral cavity.
2. Degenerative changes: That occur once the teeth have erupted and begin to wear down.

### 1) Developmental changes

#### a) HARD TISSUE FORMATION

Tooth formation begins at a very early stage of life by six month. The sequence of formation and eruption of teeth with give a precise age estimation method. In this method, each tooth is scored based on its developmental stage and scores are compared with values corresponding to a particular age (11).

For example: a deciduous second incisor crown that is fully formed and is recovered adjacent to a deciduous second incisor crown that is only ^3/4^ complete suggest a single individual of age less than 6 months (20). But, if complete deciduous second molar is found in association with these 2 teeth, then it may represent that there are more than 1 individual. This is used if dentition is completed and not applicable if there is missing teeth due to cases etc (13).

#### b) DENTAL ERUPTION

Human dentition has 2 eruption stages and their associated ages. To assess the age of unknown individual, we can compare the postmortem radiographs of the individual to the eruption standards produced by the Schour and Massler (11).

#### c) THIRD MOLAR ERUPTION

Third molar emergence tends to be around 17-19 years of age. This tooth has high variations, may be completely developed but impacted or it may be completely absent. Only radiograph can be the give the accurate document of this tooth (10).

#### d) DENTAL MEASUREMENT

This technique was an alternative to the qualitative assessment where the length of tooth was measured

### 2) Degenerative changes

That occurs once the teeth have erupted and begin to wear down. There is an intuitive connection between tooth wear and age, as those with more wear tend to be older. We can use volume of pulp cavity because it’s seen that the volume of pulp cavity reduces due to deposition of secondary dentine with ageing (8).

Age estimation is the important part in forensic odontology because human dentition follows a reliable and predictable developmental sequence.

## SEX DETERMINATION

Sex determination is very important subdivision of forensic odontology, which plays a major role in identification of the unknown individuals in natural disasters; chemical and nuclear bomb explosion scenarios

It can be done by four methods (11):

1. Craniofacial morphology and dimension: The morphology of the skull and mandible, pattern formed by six traits those are mastoid, supraorbital ridge, size and architecture of the skull, zygomatic extensions, nasal aperture, and mandible gonial angle and Frontal sinus dimension are taken into consideration.
2. Sex difference in tooth dimension: Sex determination by measuring mesiodistal and buccolingual dimensions is most simple and reliable method for sex determination. Both the dimensions are more in male than in female.
3. Tooth morphology: In male, the distal accessory ridge in canines is more prominent than in female. In female, there is less number of cusps in mandibular first molar (distobuccal or distal). These features can be because of evolutionary reduction in the female lower jaw size.
4. Sex determination by DNA analysis: The study by Das and his associates stated that the sex determination could be obtained from the studying the X and Y-chromosomes upto four weeks of the death.

## BITE MARK

The bite mark is defined as the physical alteration in or on a medium caused by the contact of teeth. In few of criminal cases it is seen that suspect or victim has left his or her teeth marks on another person or inanimate object (4). The concept of bite mark evidence is interesting and is there from Roman times.

There will be an outer edge of arches along with series of abrasion, with or without laceration that reflects the size, shape and arrangement of class characteristics of incisal or occlusal surfaces of dentition (8).

- In more aggressive bites -The assailant may suck the soft tissues into the mouth so that images of palatal and incisal surfaces of teeth may appear. Bites show laceration of tissue and petechial hemorrhage’s in the center of the wound.
- In less aggressive bites -the skin may not be completely penetrated so there can be oval mark mostly of anterior teeth.

Bite marks change over a time on living as well as dead. If the bite is on living person there will be post injury changes in the tissue, where bleeding, swelling and discoloration can be seen. If the bite is on dead Person, then photographs of marks are taken with standardized technique.

### Bites on objects

If bite is present on objects such as apple, beer, chocolate etc. often yields more information because of lack of distortion of the material and we can obtain a good impression of biting edges. Taking swabs from this object is very necessary because it may reveal the blood group is the assailant and DNA analysis is possible (13).

### Lip print

Cheiloscopy is a forensic investigation that deals with identification of human based on lip traces. Lip print wrinkle pattern has individual characteristics same as finger prints (20). The wrinkles and grooves on the labial mucosa form a characteristic pattern called lip prints. The presence or absence of a person from the crime can be verified based on lip prints since the lip prints being uniform throughout the life. The 1967 Santos was the first person to classify lip grooves. There are four types of lip grooves (13).

- Straight line
- Curved line
- Angled line
- Sine shaped line

For collection, development and recording of lip prints a uniform and standard procedure has to be developed which helps ensuring comparison.

## CONCLUSION

This article aims to provide an overview into the importance of forensic odontology with an emphasis various methods how to do so. DNA profiling provides information regarding physical characteristics, ethnicity and sex determination. Forensic odontology is that branch forensic medicine has established itself as an important and indispensable service in medico legal matters toward the creation of justice and secures society for the future inhabitants.
